# Short superficial white matter and aging: A longitudinal multi-site study of 1293 subjects and 2711 sessions

**DOI:** 10.1016/j.nbas.2023.100067

**Published:** 2023-01-16

**Authors:** Kurt G. Schilling, Derek Archer, Fang-Cheng Yeh, Francois Rheault, Leon Y. Cai, Andrea Shafer, Susan M. Resnick, Timothy Hohman, Angela Jefferson, Adam W. Anderson, Hakmook Kang, Bennett A. Landman

**Affiliations:** a Department of Radiology & Radiological Sciences, Vanderbilt University Medical Center, Nashville, TN, USA; b Vanderbilt Memory and Alzheimer’s Center, Vanderbilt University Medical Center, Nashville, TN, USA; c Department of Neurology, Vanderbilt University Medical Center, Nashville, TN, USA; d Vanderbilt Genetics Institute, Vanderbilt University School of Medicine, Nashville, TN, USA; e Department of Medicine, Vanderbilt University Medical Center, Nashville, TN, USA; f Department of Neurological Surgery, University of Pittsburgh Medical Center, Pittsburgh, PA, USA; g Department of Bioengineering, University of Pittsburgh, Pittsburgh, PA, USA; h Department of Electrical Engineering and Computer Science, Vanderbilt University, Nashville, TN, USA; i Laboratory of Behavioral Neuroscience, National Institute on Aging, National Institutes of Health, Baltimore, MD, USA; j Department of Biomedical Engineering, Vanderbilt University, Nashville, TN, USA; k Department of Biostatistics, Vanderbilt University, Nashville, TN, USA

**Keywords:** Brain aging, Superficial white matter, U-fibers, Tractography

## Abstract

It is estimated that short association fibers running immediately beneath the cortex may make up as much as 60 % of the total white matter volume. However, these have been understudied relative to the long-range association, projection, and commissural fibers of the brain. This is largely because of limitations of diffusion MRI fiber tractography, which is the primary methodology used to non-invasively study the white matter connections. Inspired by recent anatomical considerations and methodological improvements in superficial white matter (SWM) tractography, we aim to characterize changes in these fiber systems in cognitively normal aging, which provide insight into the biological foundation of age-related cognitive changes, and a better understanding of how age-related pathology differs from healthy aging. To do this, we used three large, longitudinal and cross-sectional datasets (N = 1293 subjects, 2711 sessions) to quantify microstructural features and length/volume features of several SWM systems. We find that axial, radial, and mean diffusivities show positive associations with age, while fractional anisotropy has negative associations with age in SWM throughout the entire brain. These associations were most pronounced in the frontal, temporal, and temporoparietal regions. Moreover, measures of SWM volume and length decrease with age in a heterogenous manner across the brain, with different rates of change in inter-gyri and intra-gyri SWM, and at slower rates than well-studied long-range white matter pathways. These features, and their variations with age, provide the background for characterizing normal aging, and, in combination with larger association pathways and gray matter microstructural features, may provide insight into fundamental mechanisms associated with aging and cognition.

## Introduction

Superficial white matter (SWM) is the layer of white matter immediately below the cerebral cortex, and is composed of short association fibers that may connect adjacent cortical areas (inter-gyri SWM) or run along the ridge of one gyrus (intra-gyri SWM) [1]. As summarized in [2], short association fibers represent a majority of the connections of the human brain [3], [4], occupy as much as 60 % of the total white matter volume [3], are among the last parts of the brain to myelinate [5], [6], and contain a comparatively high density of interstitial white matter neurons relative to other white matter [7], [8]. The SWM serves a critical role in brain function, plasticity, development, and aging, and is especially affected in disease and disorders [9], [10], [11], [12], [13], [14], [15], [16], [17], [18], [19], [20].

Despite its prevalence and significance, SWM has been understudied relative to the long-range association, projection, and commissural fibers of the brain. This is largely because of the limitations of diffusion MRI fiber tractography [21], [22], [23], which is the primary methodology used to non-invasively study the white matter connections [24]. The study of SWM using tractography faces anatomical and methodological challenges including partial volume effects, complex local anatomy, and a lack of consensus on definition and taxonomy [23], which complicate development and validation of algorithms dedicated to studying these fiber systems. However, recent innovation in diffusion MRI imaging, processing, and tractography methodologies [20], [21], [23], [25], [26] have made it possible to reliably study SWM in health and disease [13], [14], [15], [16], [27], [28].

One promising avenue of exploration is to study SWM during aging. Studies of the aging brain may provide insight into the biological foundation of age-related cognitive changes, and a better understanding of how abnormal aging (e.g., age-related neurodegenerative disorders) differs from healthy aging [29]. A large body of magnetic resonance imaging (MRI) research has shown that the structure of the human brain is constantly changing with age. In the gray matter, structural MRI studies have shown heterogenous patterns of normal age-related changes in cortical volume and thickness [30], [31], [32], [33], [34], [35], [36], [37], with detectable differences in abnormal aging and disease [37], [38], [39], [40], [41], [42]. In the white matter, diffusion tensor imaging (DTI) analysis has shown that fractional anisotropy (FA) is negatively associated with age and mean diffusivity (MD) is positively associated with age across several white matter pathways [43], [44], [45], [46], and tractography analysis has shown that the volume and surface areas of many pathways decreases with age [47]. These findings have been attributed to myelin loss and/or decreased axonal densities and volumes. However, with few exceptions [12], [48], [49], [50], studies of white matter brain aging have focused on the deep white matter and larger long-range pathways of the brain.

Inspired by recent anatomical considerations and methodological improvements in SWM tractography [23], and lack of studies of SWM during aging, we sought to characterize changes in these fiber systems during normal aging. To do this, we leveraged three well-established cohorts of aging, including two longitudinal cohorts [Baltimore Longitudinal Study of Aging (BLSA) [51], Vanderbilt Memory & Aging Project (VMAP) [52]], and one cross-sectional cohort [Cambridge Centre for Ageing and Neuroscience (Cam-CAN) [53]]. Within these cohorts, we performed automatic tractography segmentation in 132 SWM bundles, characterizing both microstructural features and macrostructural features of these SWM systems, to describe associations between these features and age.

## Methods

### Data

This study used data from three datasets, summarized in Table 1, and contained a total of 1293 participants (2711 sessions) aged 50–98 years. All datasets were filtered to exclude participants with diagnoses of mild cognitive impairment, Alzheimer’s disease, or dementia at baseline, or if they developed these conditions during the follow-up interval. Finally, in order to focus on the aging process, datasets were filtered to include participants aged 50+, due to limited samples sizes below 50 years old in each dataset.

**Table 1:** This study used 3 longitudinal and cross-sectional datasets, with a total of 1293 participants (2711 sessions), aged 50–98 years. Distributions of age at baseline, and number of sessions, are shown for each individual dataset.

Dataset	Number of Subjects	Number of Sessions	Age
Baltimore Longitudinal Study of Aging	741 328 M	1788 Range [1 8]	[50 98] 74.1 +/- 9.9
Cambridge Centre for Ageing Neuroscience	365 186 M	365 Range [1]	[50 88] 68.0 +/- 10.3
Vanderbilt Memory & Aging Project	187 113 M	558 Range [1 4]	[60 95] 74.2 +/- 7.0
	**1293** **627 M**	**2711** **Range [1 8]**	**[50 98]** **73.5 +/- 9.3**

First, was the Baltimore Longitudinal Study of Aging (BLSA) dataset, with 741 participants scanned multiple times ranging from 1 to 8 sessions, and time between scans ranging from 1 to 10 years, yielding a total of 1788 diffusion sessions. Diffusion MRI data was acquired on a 3 T Philips Achieva scanner (32 gradient directions, b-value = 700 s/mm2, TR/TE = 7454/75 ms, reconstructed voxel size = 0.81 × 0.81 × 2.2 mm, reconstruction matrix = 320 × 320, acquisition matrix = 115 × 115, field of view = 260 × 260 mm). Second, was data from the Vanderbilt Memory & Aging Project (VMAP), with 187 participants, scanned between 1 and 4 sessions, with a total of 558 diffusion datasets. Diffusion MRI data was acquired on a 3 T Philips Achieva scanner (32 gradient directions, b-value = 1000 s/mm2, reconstructed voxel size = 2x2x2mm). Third, was data from the Cambridge Centre for Ageing and Neuroscience (Cam-CAN) data repository [53] with 356 participants, each scanned once using a 3 T Siemens TIM Trio scanner with a 32-channel head coil (30 directions at b-value = 1000 s/mm2, 30 directions at b-value = 2000 s/mm2, reconstructed voxel size = 2x2x2mm). All datasets were preprocessed using the PreQual diffusion MRI pipeline [54], which includes motion correction, eddy current correction, and susceptibility distortion correction (using the Synb0-DISCO [55] algorithm for distortion correction for Cam-CAN and BLSA where no reverse phase encoding scans are available). Thorough manual quality control was performed, and sessions with significant artifacts (excessive motion, slice dropout, striping artifact, inadequate alignment with structural image) were removed from analysis, which included four Cam-CAN, two VMAP, and thirty-two BLSA sessions. All human datasets from Vanderbilt University were acquired after informed consent under supervision of the appropriate Institutional Review Board. This study accessed only de-identified patient information.

### Tractography and SWM bundle dissection

**Fig. 1:**
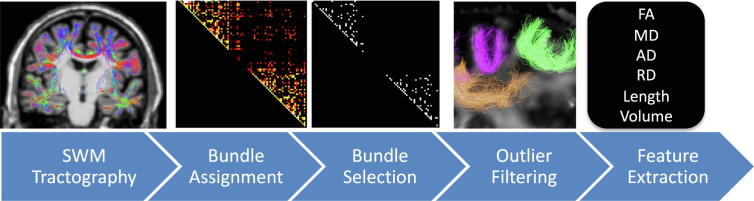
Methodological pipeline. Fiber tractography is constrained based on anatomy and length, and streamlines are assigned to edges in a connection matrix. Only bundles reproducible across the studied population (N = 132) are kept for analysis. Bundles are then filtered to remove outliers. Finally, for each bundle and each subject, microstructural and macrostructural features are extracted for analysis.

For every subject and every session, sets of SWM pathways were virtually dissected using methodology similar to [23] (referred to as ‘voxel-based’ method in [23]). Fig. 1 visualizes the methodological pipeline.

This pipeline utilized MRtrix [56]. Preprocessed diffusion data were resampled to 1 × 1 × 1 mm^3^ voxels [57] and fiber orientation distributions were derived using the 3-tissue response function estimation [58] single/multi-shell multi-tissue CSD (dependent upon the dataset) [58], [59].Alignment of diffusion and structural data was performed using a boundary based rigid registration (*epi_reg*) from the FSL toolbox [60] and subsequently quality checked for accurate alignment. Next, FreeSurfer was performed on the structural T1-weighted images [61] and FreeSurfer’s “aseg” volume was transformed to diffusioin space to act as input to MRtrix’s five tissue type (5TT) image segmentation algorithm [62]. The 5TT image was then manipulated so that cerebellar cortex, amygdala, hippocampus, and deep nuclei were set as gray matter volumes. Thus, upon creation of the white/gray matter boundary for streamline seeding all streamlines are forced to start and end at the neocortex. Tractography was performed using anatomically constrained tractography [62] and the second-order integration probabilistic algorithm [63] (max angle 45 degrees, step size = 0.5 mm, fODF power = 0.25) to generate 2 million streamlines with a maximum length of 40 mm to be consistent with the ‘short association fiber’ definition of [3] and previously validated tractography methods [23]. This pipeline has been shown to result in dense systems of fibers immediately adjacent to the cortical sheet [23].

Freesurfer [61] parcellation schemes were then transformed to diffusion MRI space. For this work, we chose to use the Desikan Killiany atlas [64] parcellation, utilizing only the neocortex labels, to assign all streamlines to edges in a connection matrix, resulting in a potential 84x84 potential SWM bundles. These bundles were filtered using scilpy tools (https://github.com/scilus/scilpy) to remove outlier streamlines using Quickbundles hierarchical clustering (alpha parameter = 0.6) [65]. An empirical decision was made to select only those bundles that are reproducible across 95 % of the studied population (containing a minimum of 500 streamlines), resulting in 132 SWM bundles studied.

While there is no consensus on taxonomy and classification of SWM [21] (just as for long range tracts [66]), we chose to visualize results of inter-gyral (connections between two different gyri, resulting in the traditionally described U-shaped fibers, or U-fibers) and intra-gyral (connections within the same gyrus, i.e. along the diagonal of the connection matrix) SWM separately. We also note that we do not necessarily constrain fibers to be immediately superficial.

A list of the 132 bundles, using nomenclature derived from the Desikan Killiany atlas, is given in the appendix.

### Feature extraction

From the final 132 bundles for each subject, 6 features were extracted including four DTI microstructural measures of fractional anisotropy (FA), and mean, radial, and axial diffusivities (MD, RD, AD) and two macrostructural measures of length and volume, following the procedures in [67], which are based on the average streamline length and volume occupied by a discretized mask of each bundle.

### Analytical plan

To investigate the relationship between age and each WM feature, linear mixed effects modeling was performed, with each (z-normalized) feature, Y, modeled as a linear function of age, $y=\beta_{0}+\beta_{1}Age+\beta_{2}Sex+{\beta_{3}TICV+\beta }_{3}\left(1+AGE\right|\mathrm{DATASET})+\beta_{4}\left(SUB\right)$, where subjects (SUB) were entered as a random effect (i.e., subject-specific random intercept), and subject sex (Sex) and total intracranial volume (TICV) as a fixed effects. Additionally, we modelled the association between age and outcome variable as dataset (DATASET) specific due to expected differences in MR protocols [68], [69], [70], [71], [72], and included a dataset specific random slope and intercept. We note that the TICV utilized was calculated from the T1-weighted image from the baseline scan.

Due to multiple comparisons, all statistical tests were controlled by the false discovery rate [73] (132 pathways × 6 features = 792 hypothesis tested) at 0.05 to determine significance. Results are presented as the beta coefficient of estimate ‘β *_1_*′, or in other words “the association of the feature ‘y’ with Age”, which (due to normalization) represents the standard deviation change in feature per year. These measures are derived for each pathway and each feature. Additionally, results may be shown as a percent change per year, derived from the slope normalized by the average value across the aging population (from 50 to 98), and multiplied by 100, which represents the percent change in feature per year. These measures are derived for each pathway and each feature.

### Comparison with long-range white matter

For comparison with the more thoroughly studied long range white matter pathways, we perform tractography and bundle segmentation using TractSeg [74] automatic segmentation resulting in 72 association, projection, and commissural bundles. Microstructure features (FA, MD, AD, RD) and macrostructure (volume, length) were extracted as done for SWM, the analyzed using the same linear mixed effects models. The purpose of this dataset is only as a benchmark for associations with age, and an in-depth exploration of the microstructural and macrostructural features of these pathways is detailed in [47] (note based on the same three datasets, although the current study has and increased number of subjects/sessions due to the longitudinal nature of the datasets).

## Results

### SWM systems

**Fig. 2:**
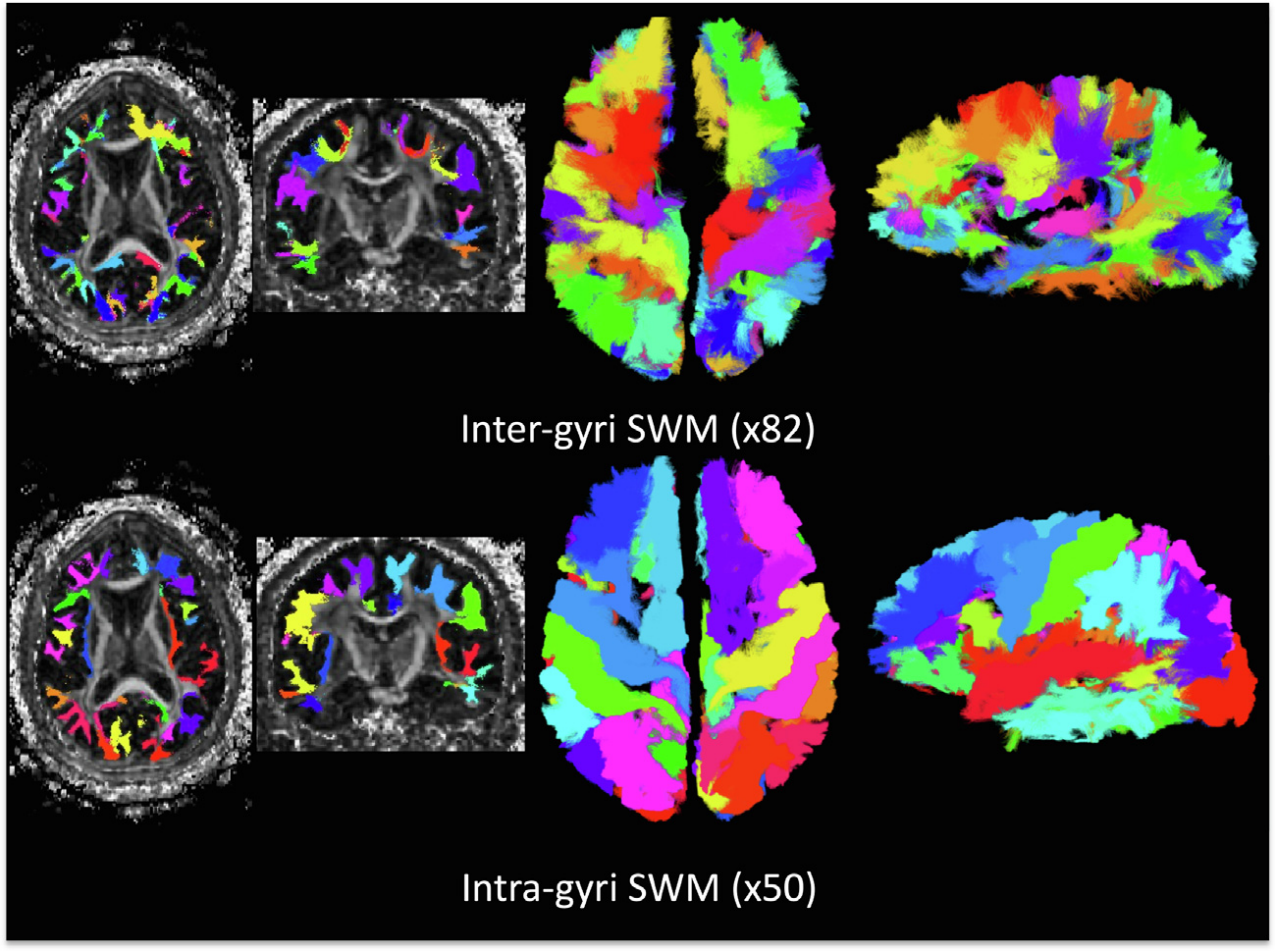
SWM systems show expected shape and locations, and cover a large portion of the surface of the brain. 132 SWM bundles determined to be robust across a population are shown in a single subject, with distinct colors for each bundle, and separated by inter-gyri and intra-gyri systems.

Example SWM systems that were consistently identified across the population are shown in Fig. 2 for a single example subject. In the coronal and axial slices, these fibers run immediately below and adjacent to the cortex in locations and geometries expected traditionally assigned to SWM. In the 3D visualization, SWM is represented along a large portion of the gray matter surface. In agreement with recent literature on tractography [23] and dissection [1], both inter-gyri and intra-gyri SWM systems exist throughout the entirety of the cortex.

### What changes and where?

**Fig. 3:**
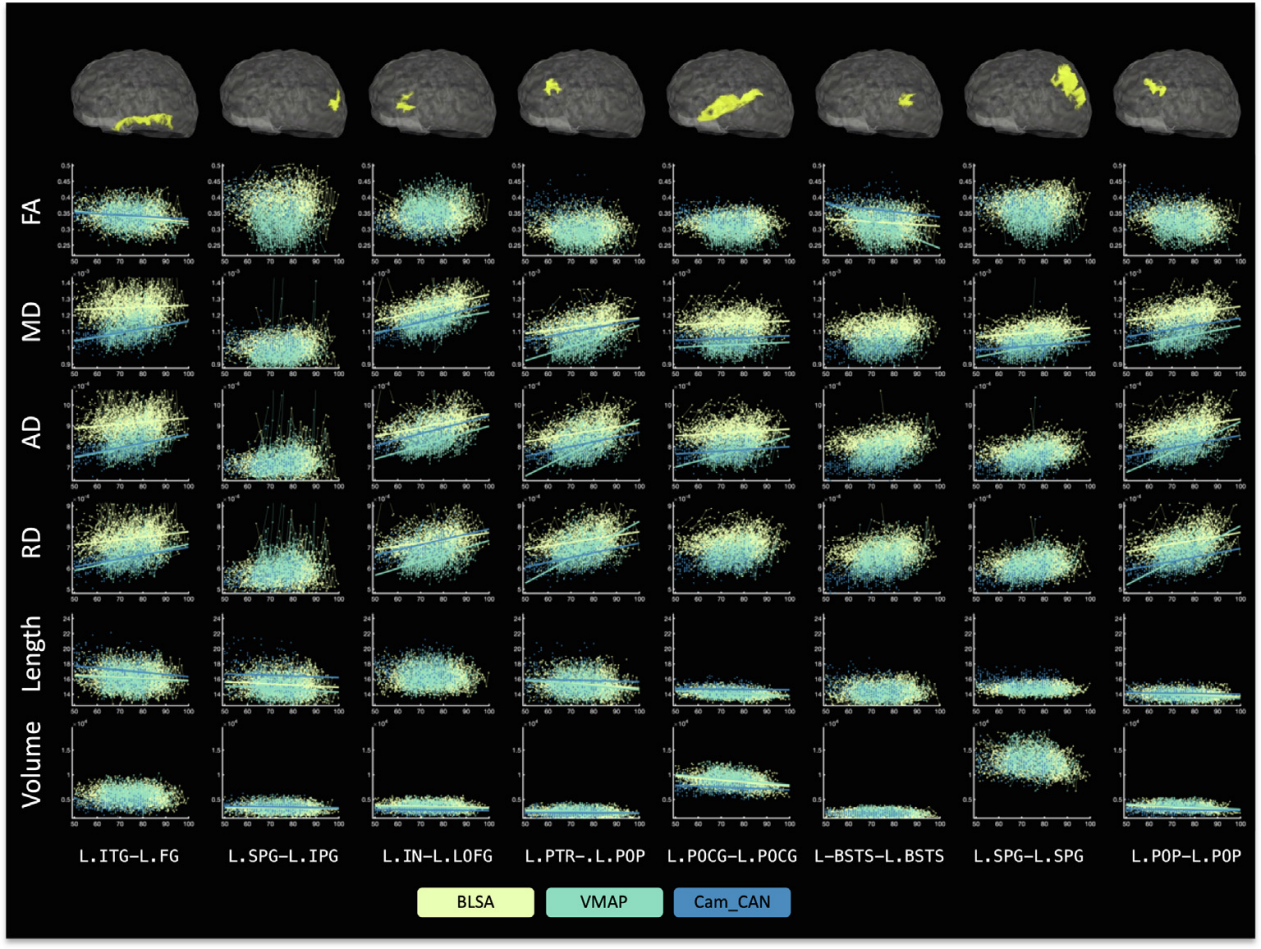
Microstructural and macrostructural features change with age in many pathways. Shown are all studied features for 8 selected pathways (4 intra-gyri, 4 inter-gyri), where all data points are shown (with lines connecting longitudinal datasets). A line of best fit is shown if there are statistically significant associations with age, where color indicates the cohort. Visualization of the SWM pathways for a single subject are shown overlaid on a transparent brain.

Fig. 3 shows associations with age of all measures for 8 selected pathways (4 intra-gyri and 4 inter-gyri systems). In line with previous literature in both long association pathways and SWM, FA frequently shows negative associations with age, while the diffusivities show positive associations with age. In general, the averaged detected streamline length and volume tend to decrease with increasing age, even when accounting for TICV, although the effects are not statistically significant for all pathways. As expected, different datasets, with different acquisitions, result in different calculated DTI indices, with much smaller differences in bundle length and volume.

**Fig. 4:**
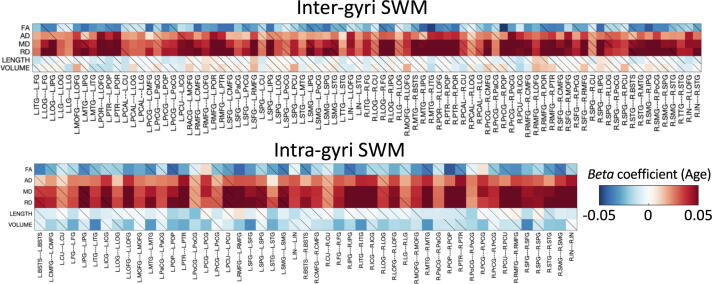
What and where changes occur in SWM during aging. The beta coefficient from linear mixed effects modeling is shown as a matrix for all features across all pathways (inter-gyri top; intra-gyri bottom). Note that the beta coefficient describes “the association of the feature ‘y’ with Age”, which (due to normalization) represents the standard deviation change in feature per year. Non-significant effects are shown as diagonal line.

**Fig. 5:**
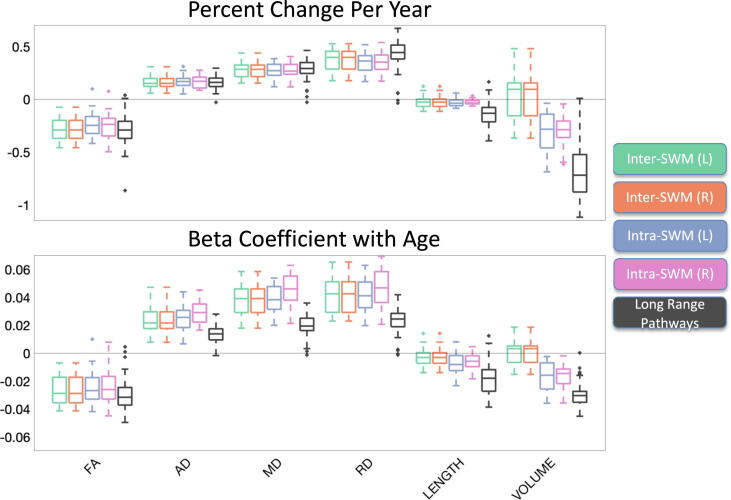
Changes in superficial white matter. The percent change per year (top) and beta coefficient (bottom) from linear mixed effects modeling across all studied SWM pathways is shown in boxplot form, for inter and intra gyri SWM, separated by hemisphere, with long-range systems described in [47] for reference. In general, diffusivities show positive associations with age, while FA, and length show negative associations with age. Association of SWM volume and age varies based on the intra/inter gyral systems.

To summarize association with age for all features and all pathways, we show the beta coefficient associations with age for all features in a matrix in Fig. 4, along with boxplots summarizing the beta coefficients and percent change with age across all studied pathways in Fig. 5. DTI measures show large, robust associations with age for many pathways. FA in SWM shows negative associations with age, while all diffusivities (AD, MD, RD) show strong positive associations with age, with similar results across intra/inter-gyri SWM and left/right hemispheres. Measures of length generally show negative associations with age, although the age effect is reduced compared to microstructure. Finally, SWM shows mixed associations with age, where inter-gyri SWM volumes have both positive and negative associations with age, with median association positive. However, intra-gyri volume consistently shows larger decreased associations with age.

Fig. 5 additionally facilitates comparisons with 72 long range white matter pathways. The relative change per year in microstructural indices of SWM white matter is similar to that of long range pathways, with decreases of −0.1 to −0.5 % change per year in FA, and increases in diffusivities of also + 0.1 to + 0.5 % per year. While the percent change is similar, the Beta coefficients (regression coefficient) is actually larger for features of diffusivity in the SWM. Finally, SWM changes in length and volume are much less than those of the long range connections.

### Visualizing change across superficial white matter

**Fig. 6:**
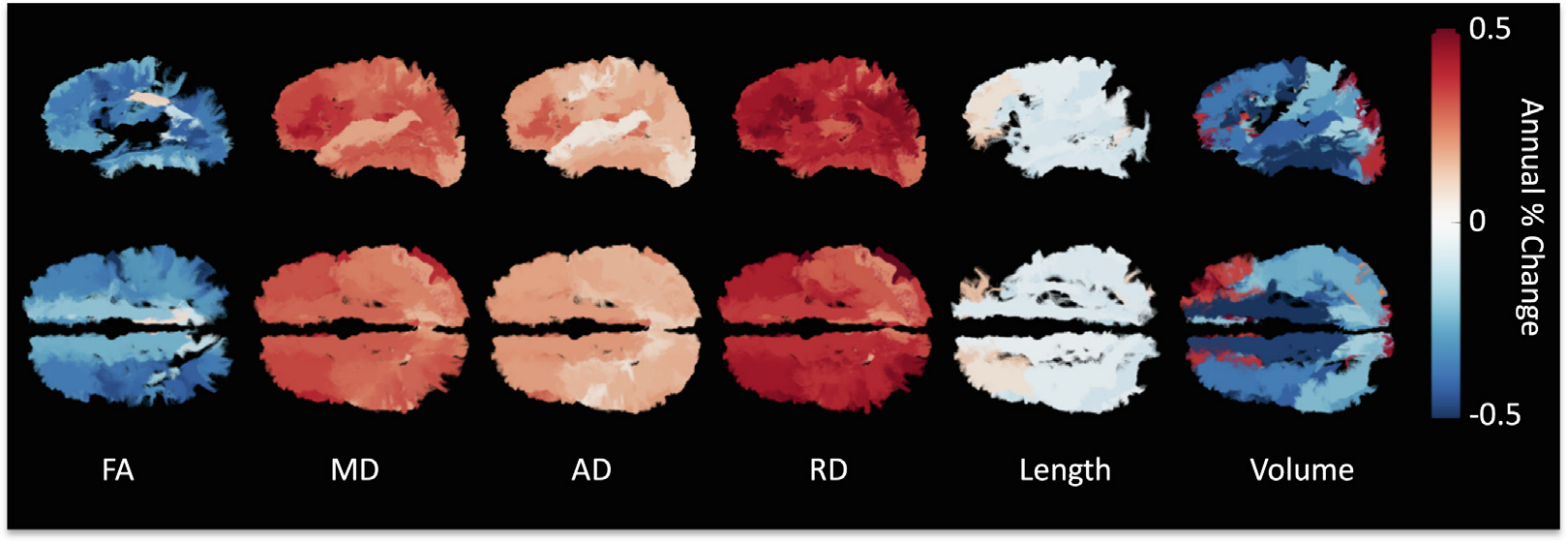
Percent change per year from the population mean shown as color-coded streamlines on an example subject. Bundles are only shown if statistically significant trends with age are observed.

To visualize where changes in SWM occur during aging, all pathways are visualized, colored coded according to percent change per year, and shown in Fig. 6. Again, SWM pathways throughout the entire cortex show statistically significant increases in diffusivities with age, of ∼ 0.1–0.45 % change per year, while FA shows decreases of similar magnitude per year. Notably, microstructural features show greatest changes in frontal and parietal lobes, with less changes in pre- and post-central gyri. Changes in length and volume are more sparse, with decreases in length with age observed throughout the entire brain, while decreases in volume with age are more heterogenous, with greater negative associations in frontal and temporal lobes.

**Fig. 7:**
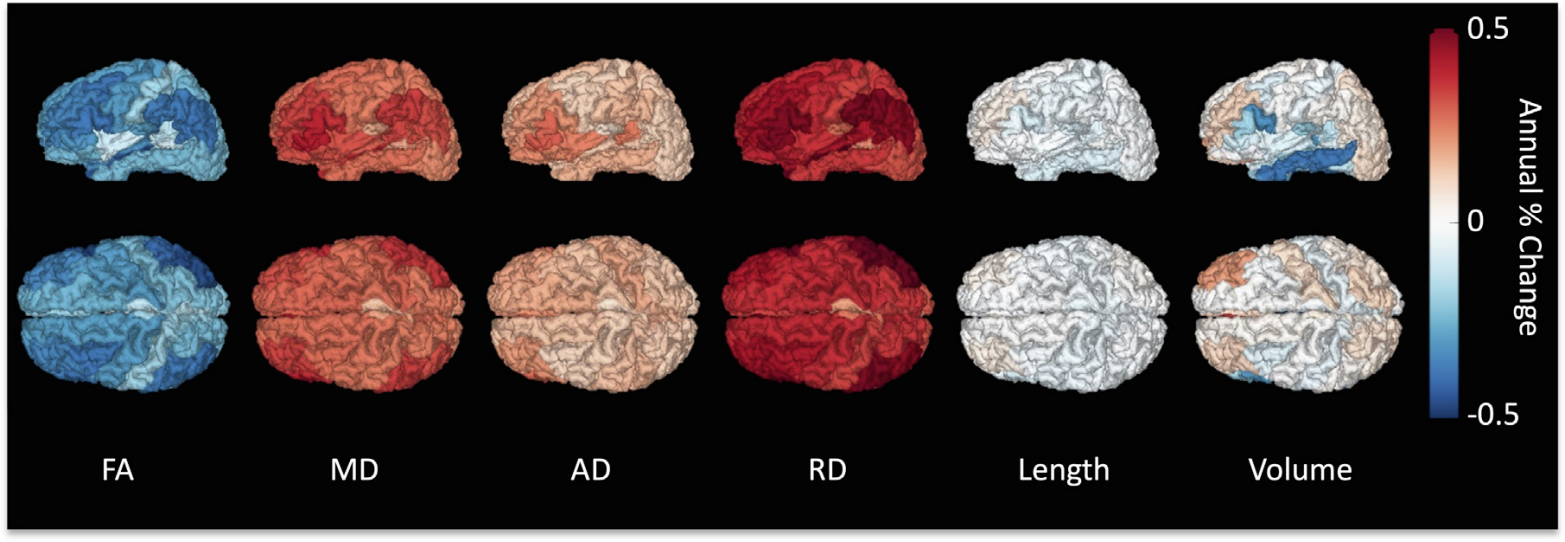
Percent change per year from the population mean for short superficial SWM connecting individual regions of interest. Regions of an example subject are color-coded based on the population-averaged percent change per year of all fibers connecting that label.

An alternative visualization is shown in Fig. 7, where each cortical region is color-coded based on the percent-change per year of all SWM fibers connecting that label (note that a single cortical region can be associated with multiple SWM systems). Again, clear patterns are observed in SWM associated with frontal and temporal lobes, including larger decreases in FA and increases in all diffusivities. Here, observed changes in volume are averaged out, with few noticeable patterns, for example averaged increase in middle and inferior frontal lobes driven by inter-gyri SWM, and decrease in inferior temporal gyrus due to intra-gyral connections.

## Discussion

Here, we have used multiple large, longitudinal and cross-sectional datasets, and innovations in tractography generation and filtering, to characterize SWM systems in 3 aging cohorts, describing microstructural features and for the first time, macrostructural features. Our main findings are that (1) diffusivities show positive associations with age, while anisotropy has negative associations with age, in SWM throughout the entire brain, (2) larger microstructural changes were observed in the frontal, temporal, and temporoparietal regions, (3) measures of SWM length decrease with age, (4) changes in volume were more heterogenous, with larger decreases in volume observed for intra-gyral SWM, and (5) microstructural of SWM have the same age associations as long-range pathways, while the volume (as derived from tractography) is less associated with age than long range-pathways.

### Superficial white matter in aging

Compared to the long-range association, projection, and commissural pathways, SWM of the brain has been underexplored in the literature, in both healthy and abnormal aging. Recently, due to advances in software and tools to study SWM, studies of these systems have started to increase. For a thorough review on SWM tractography analysis and applications, see work by Guevara et al. [21]. Of note, there have been few studies of SWM in aging using diffusion MRI. In a study of 141 healthy individuals (18–86 years old), Nazeri et al. [12] found widespread negative relationships of FA with age, in agreement with our results. To do this, they generated a population-based SWM template, and used this to perform a tract-based spatial statistics (TBSS) style analysis. Similarly, in a cohort of 65 individuals (18–74 years old) Phillips et al. [48] found age-related reductions in FA and increases in RD and AD across large areas of SWM, with results more pronounced in the frontal SWM compared to the posterior and ventral brain regions, and they interpreted this as an increased vulnerability to the aging process. Rather than tractography, this was done using white matter/gray matter surface-based alignment from structural MRI data and probing the DTI indices across the population along this boundary. Finally, using tractography and manually placed regions of interest on 69 subjects (22–84 years old), and focusing on prefrontal connections, Malykhin et al. [49] found significant decreases in FA starting at ∼ 60 years of age, in both SWM and association/commissural pathways. The use of tractography also enabled volumetric analysis, where both long range and short-range fiber systems showed decreased volumes with age.

Motivated by these works in SWM, the current study takes advantage of innovations in tractography and SWM segmentation, and incorporates multiple large cross-sectional and longitudinal cohorts totaling > 1200 participants and > 2700 sessions to study SWM throughout the entire brain. Specifically, constrained spherical deconvolution [75], in combination with probabilistic tractography [63] has become prevalent in state-of-the art studies of the human connectome and individual fiber bundles. Combining this with anatomical constraints [62] and subsequent filtering [65] enables robust delineation of white matter systems underneath most of the cortex (Fig. 1), in alignment with current knowledge of SWM. Similar methodology has been shown to result in reproducible streamlines [23], making studies of clinical cohorts plausible. Further, we include several large datasets on aging, making this the largest cohort to date to study these fibers in any clinical study.

## What changes and where

The observed associations with age include decreased FA, volume, length, and increased axial, radial, and mean diffusivities. The biological mechanism for these age-related changes is not entirely clear, due to the high sensitivity (and low specificity) of these DTI measures to various features of tissue microstructure. In general, these observations in white matter (in both health and disease) have been attributed to various biological mechanisms. Increases in radial and axial diffusivities are often associated with decreased axonal packing [76], [77], allowing for increased diffusivity in all orientations, as well as myelin thinning which may be observed as increased radial diffusivity [78], [79]. The low specificity of DTI can potentially be overcome with multi-compartment modeling, which may allow disentangling neurite densities, compartmental changes, and geometrical configurations. For example, a SWM study of individuals with young onset Alzheimer’s disease (using the white matter and gray matter boundary to define regions, as in [48]) found that these individuals exhibited decreased FA and increased diffusivities [80]. However, the use of a multi-compartment tissue model (in this case the neurite orientation dispersion and density imaging model [81], showed both a decreased neurite volume fraction and higher dispersion index, suggesting both a loss of myelinated fibers and greater dispersion (less coherent organization) of these SWM systems. While these studies were able to detect differences in extreme neurodegenerative cases, we found that these systems are sensitive in aging individuals without cognitive impairment as well. Future studies should implement similar modeling, in combination with the tractography generation and segmentation utilized in this study, to improve biological specificity of changes in healthy aging.

Identifying where changes occur during age may facilitate studying the underpinnings of cognitive and motor changes, and aid in identifying networks that are susceptible to disease and disorder. Here, much like previous studies [9], [48], [82], [83], [84], [85] in gray matter, white matter pathways, and axonal diameters, there is a clear anterior-to-posterior gradient in changes of microstructure across age. The frontal lobe is comprised of functional networks recruited for a diverse range of cognitive problems, and disruption is associated with age-related declines in cognitive processes [86]. Our study confirms that in addition to gray matter, and the larger white matter pathways, the SWM of the frontal lobe also indicate strong age-related trends. future work should investigate relationships between these neuroimaging features and age-related declines in cognition.

Another unique pattern in SWM is the difference in volume associations with age between inter and intra-gyral bundles, and differences between all SWM and long range decreases. Intra-gyral bundles have been described as running tangential to a gyrus and traversing throughout the blade [1] (see Discussion on nomenclature below). The intra-gyral SWM show a greater negative association with age than inter-gyral SWM. There are possibly many interesting interpretations of these results. First, this could be a true biological phenomenon, representing relative preservation of SWM relative to long range pathways, and further preservation of inter relative to intra-gyri SWM. The greater decreases in intra-gyri volume with age are intuitively related to increases in sulcal width (i.e. the distance between adjacent gyri) and decreases in sulcal depth with age [87] physically constraining the volume that these systems can occupy. However, there are certainly partial volume effects related to tractography (see limitations below), and partial volume effects with the thinning cortex. Nevertheless, there are measurable changes in microstructure and macrostructure of white matter nearest the cortex, that shows heterogenous across the brain.

### Towards painting a complete picture of brain aging

Noninvasive MR-imaging has slowly led to a convergence of evidence of structural and functional changes in aging. The main findings from decades of research are that the brain shrinks in overall volume and the ventricular system expands in volume [29]. The pattern of changes is heterogenous, as described here and elsewhere [29], with most analyses suggesting a 0.5 %-1% reduction in volume per year in most areas of the brain. The changes in volume are related to neuronal loss, neuronal shrinkage, decreased length of myelinated axons in white matter and reduction of synapses in the gray matter. Finally, structural changes in healthy aging mediate, or explain, domain-specific cognitive decline in individuals both with and without cognitive impairment [36], [37]. The results of this study highlight that SWM cannot be ignored when forming a complete picture of brain aging. In addition, variation of these systems across populations may enable subject-specific analysis and identification of atypical structure, which may be used to study subject-specific function.

#### Nomenclature and Taxonomy

Here, we have chosen to identify and analyze groups of SWM streamlines based on cortical connectivity defined by a commonly used parcellation scheme [64]. There are a number of ways that superficial bundles could be virtually segmented, including automated/semi-automated region placement, streamline clustering methods or latent space methods, or hybrid methodologies (see Guevara et al. [21], for a review). Much like long range connections [66] there is no clear consensus on the taxonomy and nomenclature of SWM systems, and different analysis methods and methodologies result in different bundles (see [88] for a comparison of long range white matter pathways, and [21] for a comparison of SWM systems). Our method resulted in 132 unique bundles that are reproducible across a population, in line with existing atlases or parcellation/clustering schemes with 100 SWM bundles [20], 93 SWM bundles [89], and 198 SWM bundles [90].

Recent observations using Klingler’s dissection show that in addition to the commonly observed U-fibers connecting adjacent gyri which form the thin white matter sheet of the sulcal floor, there are indeed intra-gyral SWM systems that run along the edge of gyral crowns [1]. Utilizing a simple gyral-based parcellation scheme easily allows us to classify our bundles as inter or intra-gyri. Our is the first tractography study to distinguish and analyze these systems, finding differences in their microstructural and macrostructural changes with age. Optimistically, our 132 bundles is well in line with that observed with dissections, with a range of 73–142 (mean of 97) unique superficial systems in 7 dissected hemispheres.

### Limitations and future direction

Because of the lack of studies on SWM, there are a number of research directions that can benefit from these methodologies. Understanding not only the relationship between SWM and the cortex, but also the SWM and long-range pathways would further our understanding of the complex interactions of the aging brain. Additionally, tractometry [91], [92], [93] or high dimensional analysis of the brain, which has been shown to enable single-subject inference [91], may benefit from the additional set of features provided by SWM. Understanding which features of the brain change first is paramount to understanding differences in disease. SWM has found relevant application in cohorts with autism, schizophrenia, and Alzheimer’s disease, [21] and may further benefit from a comprehensive examination of the structural changes of the brain including both white and gray matter geometric analysis and microstructure analysis. Similarly, inclusion of cognitive and motor variables will facilitate linking function to structure. Next, studies of SWM may help identify challenges for traditional fiber tractography of the long-range fibers – characterizing where these systems occur may facilitate challenges associated with gyral biases [22], [94], [95] and bottlenecks in streamline propagation that lead to creation of false positive pathways [96], [97], [98], [99]. Lastly, future studies characterizing changes in SWM together with the long range white and gray matter across the lifespan should provide quantification of variation and a benchmark of normative trajectories across a population [100].

Several limitations should be acknowledged. First, while the use of multiple longitudinal and cross sectional large datasets is particular strength of this study, the use of different datasets with different acquisitions is known to result in very different quantitative indices of microstructure and macrostructure [68], [69], [70], [71], [72]. Here, we included dataset as a variable in our mixed effects models, and consider this an advantage to the current study which shows these effects generalize across datasets. Uniquely, microstructural features showed the greatest effect sizes of dataset (both slope and intercept), although macrostructural features of length and volume for CAM-CAM did frequently show significant effects of estimated intercepts of the volume (negative effect, i.e., decreased volume), likely due to the use of a multi-shell acquisition enabling higher angular resolution and decrease partial volume effect. Alternatively, harmonization of the diffusion signal, or quantitative indices, may be used and is an active area of interest [72], [101], [102]. Second, the data used is neither high angular resolution nor high spatial resolution. The initial validation of the SWM tractography used here showed [23], [103] reliable results at comparable resolutions, although with a larger number of directions and b-values, however constrained spherical deconvolution has proven remarkably robust at estimating fiber orientation and crossing fibers even at low b-value and minimal directions [75]. Future studies should utilize higher resolution datasets (e.g., the Human Connectome Project [104]), which may reduce variability in quantification, and enable studies across the entire lifetime. Third, we chose simple linear mixed effects modelling, whereas changes across a lifespan have been shown to be nonlinear – therefore we chose to focus our analysis on age 50+. Fourth, there are several methods to segment and study SWM, both with and without tractography [20], [21], [90], [105], and we could have chosen different streamline generation and clustering algorithms. We expect that results will be similar, but not exactly the same, with the use of different methodologies for virtual dissection [88]. Next, while SWM atlases do exist [20], [25], [89], [105], [106], we choose to include all “U-shaped” fiber systems that exist within a certain percent of the studied population. This does not guarantee the existence of true anatomical connections, but has been used in the literature as an indicator of reliability of results.

A limitation of these techniques, and tractography in general, is related to partial volume effects. The process of tractography can be influenced by partial volume effects with gray matter and with other white matter systems [98] that traverse the same imaging voxel. Microstructure measures will certainly be sensitive to gray matter changes (which is known to change with age [107], [108], [109] as well as those of nearby white matter systems. Similarly, macrostructure measures of length will be highly dependent upon user-defined length thresholds [23], while volume is based on discretization of streamlines into voxels, which may be more variable for smaller SWM bundles. Because of this, measures of the intra-gyral volume may simply be a proxy for total white matter volume within a gyrus, rather than truly specific to only superficial systems. However, these changes are nonetheless interesting, and strongly associated with age. Because of these reasons, superficial white matter reproducibility is expected to be lower for superficial white matter than long-range pathways [110], particularly for low resolution and low angular resolution datasets [23]. However the streamline propagation and anatomical constraints utilized have been shown to have moderate reproducibility, with results dependent on scanner, acquisition, sampling schemes, and choices in the streamline generation process (constraints, maximum lengths, seeding, etc.) [23], a challenge that exists still in long range tractography [71]. Finally, SWM is susceptible to specificity/sensitivity tradeoffs just as long-range pathway investigations [97], [111], and anatomical validation is required in the form of tracers or cadaveric dissection, to not only verify the existence and trajectories of these pathways, but features of length and volume as well. Reassuringly, both intra- and inter-gyral SWM is visible in cadaveric samples throughout the entire cerebral hemisphere [1], just as in our results (Fig. 2).

## Conclusion

Here, we have used a large, longitudinal dataset, and innovations in tractography generation and filtering, to characterize SWM systems in an aging cohort, describing microstructural features and for the first time, macrostructural features. We find robust associations with age for all features, across many fiber systems. These features, and their normal variations with age, may be useful for characterizing abnormal aging, and, in combination with larger association pathways and gray matter microstructural features, lead to insight into fundamental mechanisms associated with aging and cognition.

## Statements and Declarations

### Funding

This work was supported by the National Science Foundation Career Award #1452485, the National Institutes of Health under award numbers R01EB017230, K01EB032898-01, and in part by ViSE/VICTR VR3029 and the National Center for Research Resources, Grant UL1 RR024975–01. VMAP data is supported by the following grants: Alzheimer’s Association IIRG-08-88733 (ALJ), R01-AG034962 (ALJ), K24-AG046373 (ALJ), UL1-TR000445 and UL1-TR002243 (Vanderbilt Clinical Translational Science Award), S10-OD023680 (Vanderbilt’s High-Performance Computer Cluster for Biomedical Research). P20-AG068082 (Vanderbilt Alzheimer’s Disease Research Center).

### Ethic Approval

All human datasets from Vanderbilt University were acquired after informed consent under supervision of the appropriate Institutional Review Board. All additional datasets are freely available and unrestricted for non-commercial research purposes. This study accessed only de-identified patient information.

### Consent to participate

Informed consent was obtained from all individual participants included in the study.

## Declaration of Competing Interest

The authors declare that they have no known competing financial interests or personal relationships that could have appeared to influence the work reported in this paper.

## Figures and Tables

**Table:** 

Inter-gyri SWM		Intra-gyri SWM	
Abbreviation	Freesurfer-based nomenclature	Abbreviation	Freesurfer-based nomenclature
L.ITG---L.FG	ctx-lh-inferiortemporal---ctx-lh-fusiform	L.LG---L.LG	ctx-lh-lingual---ctx-lh-lingual
L.LOG---L.FG	ctx-lh-lateraloccipital---ctx-lh-fusiform	L.BSTS---L.BSTS	ctx-lh-bankssts---ctx-lh-bankssts
L.LOG---L.IPG	ctx-lh-lateraloccipital---ctx-lh-inferiorparietal	L.CMFG---L.CMFG	ctx-lh-caudalmiddlefrontal---ctx-lh-caudalmiddlefrontal
L.LG---L.LOG	ctx-lh-lingual---ctx-lh-lateraloccipital	L.CU---L.CU	ctx-lh-cuneus---ctx-lh-cuneus
L.MOFG---L.LOFG	ctx-lh-medialorbitofrontal---ctx-lh-lateralorbitofrontal	L.FG---L.FG	ctx-lh-fusiform---ctx-lh-fusiform
L.MTG---L.IPG	ctx-lh-middletemporal---ctx-lh-inferiorparietal	L.IPG---L.IPG	ctx-lh-inferiorparietal---ctx-lh-inferiorparietal
L.MTG---L.ITG	ctx-lh-middletemporal---ctx-lh-inferiortemporal	L.ITG---L.ITG	ctx-lh-inferiortemporal---ctx-lh-inferiortemporal
L.POR---L.LOFG	ctx-lh-parsorbitalis---ctx-lh-lateralorbitofrontal	L.ICG---L.ICG	ctx-lh-isthmuscingulate---ctx-lh-isthmuscingulate
L.PTR---L.POP	ctx-lh-parstriangularis---ctx-lh-parsopercularis	L.LOG---L.LOG	ctx-lh-lateraloccipital---ctx-lh-lateraloccipital
L.PTR---L.POR	ctx-lh-parstriangularis---ctx-lh-parsorbitalis	L.LOFG---L.LOFG	ctx-lh-lateralorbitofrontal---ctx-lh-lateralorbitofrontal
L.PCAL---L.CU	ctx-lh-pericalcarine---ctx-lh-cuneus	L.MOFG---L.MOFG	ctx-lh-medialorbitofrontal---ctx-lh-medialorbitofrontal
L.PCAL---L.LOG	ctx-lh-pericalcarine---ctx-lh-lateraloccipital	L.MTG---L.MTG	ctx-lh-middletemporal---ctx-lh-middletemporal
L.PCAL---L.LG	ctx-lh-pericalcarine---ctx-lh-lingual	L.PaCG---L.PaCG	ctx-lh-paracentral---ctx-lh-paracentral
L.PrCG---L.CMFG	ctx-lh-precentral---ctx-lh-caudalmiddlefrontal	L.POP---L.POP	ctx-lh-parsopercularis---ctx-lh-parsopercularis
L.PrCG---L.PaCG	ctx-lh-precentral---ctx-lh-paracentral	L.PTR---L.PTR	ctx-lh-parstriangularis---ctx-lh-parstriangularis
L.PrCG---L.POP	ctx-lh-precentral---ctx-lh-parsopercularis	L.PoCG---L.PoCG	ctx-lh-postcentral---ctx-lh-postcentral
L.PrCG---L.PoCG	ctx-lh-precentral---ctx-lh-postcentral	L.PCG---L.PCG	ctx-lh-posteriorcingulate---ctx-lh-posteriorcingulate
L.PCU---L.ICG	ctx-lh-precuneus---ctx-lh-isthmuscingulate	L.PrCG---L.PrCG	ctx-lh-precentral---ctx-lh-precentral
L.RACG---L.MOFG	ctx-lh-rostralanteriorcingulate---ctx-lh-medialorbitofrontal	L.PCU---L.PCU	ctx-lh-precuneus---ctx-lh-precuneus
L.RMFG---L.CMFG	ctx-lh-rostralmiddlefrontal---ctx-lh-caudalmiddlefrontal	L.RMFG---L.RMFG	ctx-lh-rostralmiddlefrontal---ctx-lh-rostralmiddlefrontal
L.RMFG---L.LOFG	ctx-lh-rostralmiddlefrontal---ctx-lh-lateralorbitofrontal	L.SFG---L.SFG	ctx-lh-superiorfrontal---ctx-lh-superiorfrontal
L.RMFG---L.POR	ctx-lh-rostralmiddlefrontal---ctx-lh-parsorbitalis	L.SPG---L.SPG	ctx-lh-superiorparietal---ctx-lh-superiorparietal
L.RMFG---L.PTR	ctx-lh-rostralmiddlefrontal---ctx-lh-parstriangularis	L.STG---L.STG	ctx-lh-superiortemporal---ctx-lh-superiortemporal
L.SFG---L.CMFG	ctx-lh-superiorfrontal---ctx-lh-caudalmiddlefrontal	L.SMG---L.SMG	ctx-lh-supramarginal---ctx-lh-supramarginal
L.SFG---L.PaCG	ctx-lh-superiorfrontal---ctx-lh-paracentral	L.IN---L.IN	ctx-lh-insula---ctx-lh-insula
L.SFG---L.PrCG	ctx-lh-superiorfrontal---ctx-lh-precentral	R.BSTS---R.BSTS	ctx-rh-bankssts---ctx-rh-bankssts
L.SFG---L.RMFG	ctx-lh-superiorfrontal---ctx-lh-rostralmiddlefrontal	R.CMFG---R.CMFG	ctx-rh-caudalmiddlefrontal---ctx-rh-caudalmiddlefrontal
L.SPG---L.CU	ctx-lh-superiorparietal---ctx-lh-cuneus	R.CU---R.CU	ctx-rh-cuneus---ctx-rh-cuneus
L.SPG---L.IPG	ctx-lh-superiorparietal---ctx-lh-inferiorparietal	R.FG---R.FG	ctx-rh-fusiform---ctx-rh-fusiform
L.SPG---L.LOG	ctx-lh-superiorparietal---ctx-lh-lateraloccipital	R.IPG---R.IPG	ctx-rh-inferiorparietal---ctx-rh-inferiorparietal
L.SPG---L.PoCG	ctx-lh-superiorparietal---ctx-lh-postcentral	R.ITG---R.ITG	ctx-rh-inferiortemporal---ctx-rh-inferiortemporal
L.SPG---L.PCU	ctx-lh-superiorparietal---ctx-lh-precuneus	R.ICG---R.ICG	ctx-rh-isthmuscingulate---ctx-rh-isthmuscingulate
L.STG---L.MTG	ctx-lh-superiortemporal---ctx-lh-middletemporal	R.LOG---R.LOG	ctx-rh-lateraloccipital---ctx-rh-lateraloccipital
L.SMG---L.IPG	ctx-lh-supramarginal---ctx-lh-inferiorparietal	R.LOFG---R.LOFG	ctx-rh-lateralorbitofrontal---ctx-rh-lateralorbitofrontal
L.SMG---L.PoCG	ctx-lh-supramarginal---ctx-lh-postcentral	R.LG---R.LG	ctx-rh-lingual---ctx-rh-lingual
L.SMG---L.SPG	ctx-lh-supramarginal---ctx-lh-superiorparietal	R.MOFG---R.MOFG	ctx-rh-medialorbitofrontal---ctx-rh-medialorbitofrontal
L.SMG---L.STG	ctx-lh-supramarginal---ctx-lh-superiortemporal	R.MTG---R.MTG	ctx-rh-middletemporal---ctx-rh-middletemporal
L.TTG---L.STG	ctx-lh-transversetemporal---ctx-lh-superiortemporal	R.PaCG---R.PaCG	ctx-rh-paracentral---ctx-rh-paracentral
L.IN---L.LOFG	ctx-lh-insula---ctx-lh-lateralorbitofrontal	R.POP---R.POP	ctx-rh-parsopercularis---ctx-rh-parsopercularis
L.IN---L.STG	ctx-lh-insula---ctx-lh-superiortemporal	R.PTR---R.PTR	ctx-rh-parstriangularis---ctx-rh-parstriangularis
R.ITG---R.FG	ctx-rh-inferiortemporal---ctx-rh-fusiform	R.PoCG---R.PoCG	ctx-rh-postcentral---ctx-rh-postcentral
R.LOG---R.CU	ctx-rh-lateraloccipital---ctx-rh-cuneus	R.PCG---R.PCG	ctx-rh-posteriorcingulate---ctx-rh-posteriorcingulate
R.LOG---R.FG	ctx-rh-lateraloccipital---ctx-rh-fusiform	R.PrCG---R.PrCG	ctx-rh-precentral---ctx-rh-precentral
R.LOG---R.IPG	ctx-rh-lateraloccipital---ctx-rh-inferiorparietal	R.PCU---R.PCU	ctx-rh-precuneus---ctx-rh-precuneus
R.LG---R.LOG	ctx-rh-lingual---ctx-rh-lateraloccipital	R.RMFG---R.RMFG	ctx-rh-rostralmiddlefrontal---ctx-rh-rostralmiddlefrontal
R.MOFG---R.LOFG	ctx-rh-medialorbitofrontal---ctx-rh-lateralorbitofrontal	R.SFG---R.SFG	ctx-rh-superiorfrontal---ctx-rh-superiorfrontal
R.MTG---R.BSTS	ctx-rh-middletemporal---ctx-rh-bankssts	R.SPG---R.SPG	ctx-rh-superiorparietal---ctx-rh-superiorparietal
R.MTG---R.IPG	ctx-rh-middletemporal---ctx-rh-inferiorparietal	R.STG---R.STG	ctx-rh-superiortemporal---ctx-rh-superiortemporal
R.MTG---R.ITG	ctx-rh-middletemporal---ctx-rh-inferiortemporal	R.SMG---R.SMG	ctx-rh-supramarginal---ctx-rh-supramarginal
R.POR---R.LOFG	ctx-rh-parsorbitalis---ctx-rh-lateralorbitofrontal	R.IN---R.IN	ctx-rh-insula---ctx-rh-insula
R.PTR---R.POP	ctx-rh-parstriangularis---ctx-rh-parsopercularis		
R.PTR---R.POR	ctx-rh-parstriangularis---ctx-rh-parsorbitalis		
R.PCAL---R.CU	ctx-rh-pericalcarine---ctx-rh-cuneus		
R.PCAL---R.LOG	ctx-rh-pericalcarine---ctx-rh-lateraloccipital		
R.PCAL---R.LG	ctx-rh-pericalcarine---ctx-rh-lingual		
R.PrCG---R.CMFG	ctx-rh-precentral---ctx-rh-caudalmiddlefrontal		
R.PrCG---R.PaCG	ctx-rh-precentral---ctx-rh-paracentral		
R.PrCG---R.POP	ctx-rh-precentral---ctx-rh-parsopercularis		
R.PrCG---R.PoCG	ctx-rh-precentral---ctx-rh-postcentral		
R.PCU---R.ICG	ctx-rh-precuneus---ctx-rh-isthmuscingulate		
R.RMFG---R.CMFG	ctx-rh-rostralmiddlefrontal---ctx-rh-caudalmiddlefrontal		
R.RMFG---R.LOFG	ctx-rh-rostralmiddlefrontal---ctx-rh-lateralorbitofrontal		
R.RMFG---R.POR	ctx-rh-rostralmiddlefrontal---ctx-rh-parsorbitalis		
R.RMFG---R.PTR	ctx-rh-rostralmiddlefrontal---ctx-rh-parstriangularis		
R.SFG---R.CMFG	ctx-rh-superiorfrontal---ctx-rh-caudalmiddlefrontal		
R.SFG---R.MOFG	ctx-rh-superiorfrontal---ctx-rh-medialorbitofrontal		
R.SFG---R.PrCG	ctx-rh-superiorfrontal---ctx-rh-precentral		
R.SFG---R.RMFG	ctx-rh-superiorfrontal---ctx-rh-rostralmiddlefrontal		
R.SPG---R.CU	ctx-rh-superiorparietal---ctx-rh-cuneus		
R.SPG---R.IPG	ctx-rh-superiorparietal---ctx-rh-inferiorparietal		
R.SPG---R.LOG	ctx-rh-superiorparietal---ctx-rh-lateraloccipital		
R.SPG---R.PoCG	ctx-rh-superiorparietal---ctx-rh-postcentral		
R.SPG---R.PCU	ctx-rh-superiorparietal---ctx-rh-precuneus		
R.STG---R.BSTS	ctx-rh-superiortemporal---ctx-rh-bankssts		
R.STG---R.MTG	ctx-rh-superiortemporal---ctx-rh-middletemporal		
R.SMG---R.IPG	ctx-rh-supramarginal---ctx-rh-inferiorparietal		
R.SMG---R.PoCG	ctx-rh-supramarginal---ctx-rh-postcentral		
R.SMG---R.SPG	ctx-rh-supramarginal---ctx-rh-superiorparietal		
R.SMG---R.STG	ctx-rh-supramarginal---ctx-rh-superiortemporal		
R.TTG---R.STG	ctx-rh-transversetemporal---ctx-rh-superiortemporal		
R.IN---R.LOFG	ctx-rh-insula---ctx-rh-lateralorbitofrontal		
R.IN---R.STG	ctx-rh-insula---ctx-rh-superiortemporal		

## Data Availability

Derived microstructure and macrostructure features, for all pathways and subjects, along with demographic information, are made available at (link upon acceptance) for VMAP and CAMCAN datasets. Data from the BLSA are available on request from the BLSA website (https://blsa.nih.gov). All requests are reviewed by the BLSA Data Sharing Proposal Review Committee and may also be subject to approval from the NIH institutional review board.
